# Oxidative Stress and Inflammation in Heart Disease: Do Antioxidants Have a Role in Treatment and/or Prevention?

**DOI:** 10.4061/2011/514623

**Published:** 2011-08-11

**Authors:** Fredric J. Pashkow

**Affiliations:** ^1^John A. Burns School of Medicine, University of Hawai‘i, Honolulu, HI, USA; ^2^Manoa Innovation Center, 2800 Woodlawn Drive, Honolulu, HI 96822, USA

## Abstract

Inflammation triggered by oxidative stress is the cause of much, perhaps even most, chronic human disease including human aging. The oxidative stress originates mainly in mitochondria from reactive oxygen and reactive nitrogen species (ROS/RNS) and can be identified in most of the key steps in the pathophysiology of atherosclerosis and the consequential clinical manifestations of cardiovascular disease. In addition to the formation of atherosclerosis, it involves lipid metabolism, plaque rupture, thrombosis, myocardial injury, apoptosis, fibrosis and failure. The recognition of the critical importance of oxidative stress has led to the enthusiastic use of antioxidants in the treatment and prevention of heart disease, but the results of prospective, randomized clinical trials have been overall disappointing. Can this contradiction be explained and what are its implications for the discovery/development of future antioxidant therapeutics?

## 1. Introduction

While the importance of inflammation in illnesses where the phenomenon is overt, such as following trauma or infection has been recognized since ancient times, its presence and crucial role in the manifestation of many diseases never previously recognized as inflammatory is relatively recent. In such instances, the source of the inflammation is also often imperceptible [1]. This is especially relevant to the many pervasive chronic diseases that are still responsible for so much human suffering. We are currently achieving a major understanding of what is involved in the initiation of the inflammatory signaling cascade as well as the complex signaling pathways themselves that transcribe and counterregulate the molecular messengers (cytokines) that generate the biological combatants such as the inflammatory enzymes associated with the numerous relevant pathologies. In this paper we will overview some of the details of our emerging understanding of inflammation and its principal source, oxidative stress. Further, we will critically review the historical advocacy of antioxidants for the treatment and prevention of inflammatory-initiating oxidative stress, and proffer explanations for the failure to date of antioxidants to achieve therapeutic success. Finally, we will discuss the appropriateness of oxidative stress as a therapeutic target in cardiovascular disease [2] and the implications this has in us moving forward in the discovery and development of new safe and effective cardiovascular drugs.

## 2. Inflammation: A Major Cause of Human Disease

While inflammation occurring as a consequence of oxidative stress is not the only biological manifestation of excess ROS/RNS [3], inflammation resulting from oxidative stress is the cause of much human disease [4]. Typical examples are dyslipidemia [5], thrombosis [6, 7], metabolic syndrome [8], type 2 diabetes [9], nonalcoholic steatohepatitis (NASH) [10–12], macular degeneration [13], and neurodegenerative diseases such as Alzheimer's [14]. Inflammation is also a key factor in all aspects of coronary disease including the initiation and progression of atherosclerotic plaque, plaque rupture, and thrombosis (atherothrombosis), especially in recurrent thrombosis where oxidative stress is known to play a significant role [15] (Figure 4), including in those with normal cholesterol levels and in those being treated with “statins” and antiplatelet agents. This inflammation, caused by oxidative stress, could be a target for a great next wave of cardiovascular therapeutics.

## 3. Role of Oxidative Stress

Oxidative stress has been identified as critical in most of the key steps in the pathophysiology of atherosclerosis and acute thrombotic events, including dyslipidemia leading to atheroma formation, the oxidation of LDL, endothelial dysfunction, plaque rupture, myocardial ischemic injury, and recurrent thrombosis (i.e., the secondary, or subsequent clot that often occurs after initial thrombolysis). The role of oxidative stress in the connection between the various coronary disease risk factors such as elevated blood pressure, diabetes and cigarette smoking, and the clinical sequelae of disease associated with vasoconstriction, thrombosis, plaque rupture, and vascular remodeling has been recognized by Moreno and Fuster [22] but its recognition as a specific therapeutic target represents an elevation in its importance in the oxidative stress hypothesis [23]. Proinflammatory cytokines are also involved in cardiac muscle dysfunction and in the complex syndrome of heart failure [24–27]. Oxidative stress has been implicated as well in diabetic cardiomyopathy [28, 29], congestive cardiomyopathy [30], and hypertensive heart disease [31].

## 4. Potential Role of Antioxidants

Recently, progress has been made regarding the source of the oxidative stress and an understanding has been achieved regarding the role of the signaling cascade that moderates the resulting inflammatory process. However, as far back as the late 1940's (and perhaps before), antioxidants such as vitamin E have been suggested as potentially useful in the treatment of vascular disease [32]. Studies on the inhibition of experimental cholesterol arteriosclerosis in animals were published around 1949-1950 and specific discussions of the use of vitamin E in the treatment of cardiovascular disease appeared the same year [33, 34]. 

Over the years an oxidative stress hypothesis supported by epidemiologic and observational evidence that encouraged belief in and the use of antioxidants [35, 36]. For example, studies of fruit and vegetable consumption, those particularly rich in vitamin C and other antioxidants, correlated with a reduction in CVD mortality [37]. Further, the plasma level of vitamin E was inversely related to mortality from ischemic heart disease [38]. Numerous observational studies, such as the Nurses' Health Study, reported significantly reduced risk in those taking vitamin E [39].

## 5. Human Proof-of-Concept Studies Initially Encouraging

Up to the year 2000, several “smallish” trials using various combinations of antioxidant vitamins and drugs were reported as “positive.” This produced optimism in the community of antioxidant advocates. The conclusions from a selection of these Phase 2-type studies are summarized in the remainder of this paragraph and Table 1. In hemodialysis patients with prevalent cardiovascular disease, supplementation with 800 IU/day vitamin E reduced composite cardiovascular disease endpoints and myocardial infarction according to Boaz and colleagues in Israel [16]. Singh and associates reported results from a study in India suggesting that combined treatment with antioxidant vitamins A, E, C, and beta-carotene in patients with recent acute myocardial infarction might be protective against cardiac necrosis and oxidative stress and could be beneficial in preventing complications and in reduction of the cardiac event rate in ACS patients [17]. Chamiec and colleagues reported results supporting the hypothesis that in patients with AMI, oxygen-free radical-induced cellular damage contributes to alterations in electric function of the heart as seen on the signal-averaged ECG (SAECGs), and that vitamins C and E could reduce these alterations [18]. Yokoi et al. reported that probucol administered beginning 4 weeks before PTCA appears to reduce subsequent restenosis rates [19]. Salonen et al. reported that combined supplementation with reasonable doses of both vitamin E and slow-release vitamin C could retard the progression of common carotid atherosclerosis in men [20]. And finally, Tardif and his colleagues reported from Montreal that the antioxidant probucol, with or without a combination of antioxidant vitamins, is effective in reducing the rate of restenosis after balloon coronary angioplasty [21].

It is important to note that these studies are all fairly modest in size with the exception of ASAP [20] which enrolled more than 500 subjects, and that with the exception of SPACE [16], use a surrogate measure for the primary endpoint. Note that all of the therapies tested, again with the exception of SPACE, involved subgroups where more than one antioxidant or combinations of therapy were used. Finally, while the statistical analyses suggest overall significance of the studies' findings, only those receiving the drug probucol with or without multivitamins demonstrated significant effect in the Mutivitamins and Probucol Study Group [21]. The implications of these observations will be discussed further below.

## 6. Larger Randomized Clinical Trials Unsupportive

**Figure 1:**
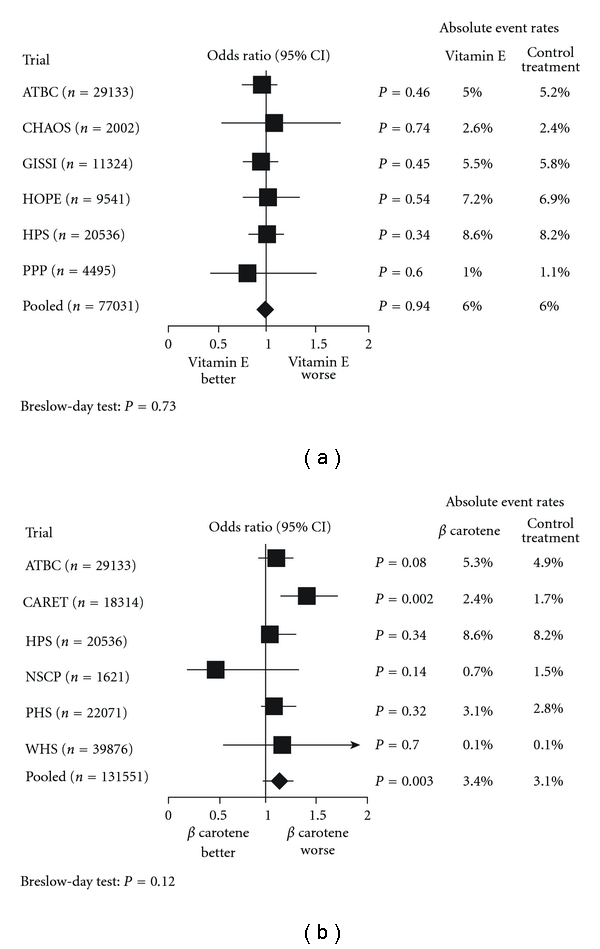
(a) Meta-analysis of large randomized trials of vit E versus placebo. Meta-analysis of 7 randomized trials involving 77,031 patients comparing the risk of cardiovascular death among those randomized to placebo or vitamin E (Breslow-Day test, *P* = 0.73). ATBC: *α*-Tocopherol, *β*-Carotene Cancer Prevention trial; CHAOS: Cambridge Heart Antioxidant Study; CI: Confidence Interval; GISSI – Gruppo Italiano per lo Studio della Sopravivenza nell'Infarto; HOPE: Heart Ourcomes Prevention Evaluation; HPS: Heart Protection Study; PPP: Primary Prevention Project, modified from [40]. (b) Meta-analysis of Randomized Trials of *β*-carotene versus Placebo. Meta-analysis of 6 randomized trials involving 131,551 patients comparing the risk of cardiovascular death among those randomized to placebo or *β*-carotene (Breslow-Day test, *P* = 0.12). ATBC: *α*-Tocopherol, *β*-Carotene Cancer Prevention trial; CARET: *β*-Carotene and Retinol Efficacy Trial; HPS: Heart Protection Study; NSCP: Nambour Skin Cancer Prevention; PHS: Physicians' Health Study; WHS: Women's Health Study, modified from [40].

But problems developed with the performance of larger randomized clinical trials [41]. An earlier meta-analysis of 6 large (>1,000 subjects) randomized trials of vitamin E with pooled data from over 77,000 subjects and 6 trials of *β*-carotene in over 131,000 subjects showed that the use of vitamin E was a “wash” (*P* = 0.94) (Figure 1(a)) and that *β*-carotene use was associated with a worse outcome (*P* = 0.003) (Figure 1(b)) [40].

The Cambridge Heart Antioxidant Study (CHAOS) buoyed hopes for believers in the oxidant stress hypothesis when it demonstrated a significant reduction in nonfatal MI (*P* = 0.0001) but offsetting that finding was an insignificant difference in cardiovascular deaths (*P* = 0.78) [42]. However, in a large, long-term trial of male physicians, neither vitamin E nor vitamin C supplementation reduced the risk of major cardiovascular events [43]. In women at high risk for CVD there were no overall effects of ascorbic acid, vitamin E or *β*-carotene on cardiovascular events [44]. Hence, there is a need for a better understanding and more scientific evidence of the relative contribution of major nutraceutical constituents to the inhibition of the progression of atherosclerosis and its clinical consequences [45].

Studies looking at progression of atherosclerosis using vitamins E, C or *β*-carotene have also been inconclusive [47]. However, certain specific patient populations or clinical circumstances showed promise: for example, reduction in the development of transplant atherosclerosis [48]. In acute coronary syndrome, N-Acetylcysteine (NAC) appeared to produce a statistically significant improvement in cardiac index in STEMI patients treated with streptokinase and NTG [49], and in a more recent study, a significant reduction of in-hospital deaths in patients undergoing primary PCI [50].

## 7. Why the Failure of So Many Antioxidant Trials?

The question is, if oxidative stress is so critical in the development and manifestations of coronary heart disease, why is it that so many of the larger antioxidant trials have failed [52]? Before specifically attempting to answer this critical question, let us go back to basics for a moment. The utilization of oxygen as an integral part of the process for generating metabolic energy (i.e., mainly via the Electron Transport Chain or ETC on the inner membrane of the mitochondria) produces reactive oxygen species (ROS) [53, 54]. These reactive oxygen species can damage cells or components of cells by oxidizing DNA or proteins or starting chemical chain reactions such as lipid peroxidation which incidentally, occurs mainly *inside* the bilayer membrane of cells, nuclei, and mitochondria [55]. ROS can be quite destabilizing to membrane integrity, but they do have important useful functions, such as the maintenance of balanced intracellular redox signaling. The function of antioxidant systems is not to remove these oxidants entirely, but instead to keep them at a level below which they will trigger the inflammatory cascade, a series of intracellular and intranuclear signaling that results in the release of destructive inflammatory cytokines [56, 57].

## 8. Inflammation Is Complex

Pathological inflammation, a complex whole-cellular pathway, is a cascade that begins with the production of excess free radicals that frequently arise from mitochondria responding to internal or environmental stress and that trigger several signaling steps that endup producing the substances that actually cause the classical signs of redness, swelling, and pain in inflammation [1]. NF-kappa-B, one of the key signaling molecules, is a transcription factor that upregulates the production of downstream inflammatory mediators, including tumor necrosis factor-alpha (TNF-alpha) [58], inducible NO synthase (iNOS), cyclooxygenase-2 (COX-2), and interleukein-1beta (IL-1beta). Recent studies have suggested that CD40-CD40L interactions themselves regulate oxidative stress and affect various signaling pathways in both the immunological and cardiovascular systems [59]. Normal cellular functions “suckup” these unwanted ROS allowing the signaling molecules, or cellular pathways such as NF-kappa-B, to operate normally along with the downstream products of these pathways, creating a balanced ROS environment and normal cellular health.

## 9. Erroneous Assumptions Influenced Trial Design

So why have so many antioxidant therapies failed when tested in randomized clinical trials? Studies were conducted based upon epidemiologic findings of benefit from surveys documenting increased intake of dietary fruits/vegetables [60]. The well-known biases of such studies aside, the assumption has been that “known” nutritional compounds that is, vitamin E, vitamin C, and *β*-carotene, are mainly responsible for the benefit. The favorable effects shown by some studies relating antioxidant dietary intake and cardiovascular disease may have been exerted by other chemicals present in foods. Flavonoids, for example, are ideal candidates, since they are plentiful in foods containing antioxidant vitamins (i.e., fruits and vegetables) and are potent antioxidants [61]. Other examples include cocoa [62, 63] and the active ingredients in wine [64, 65].

**Figure 2:**
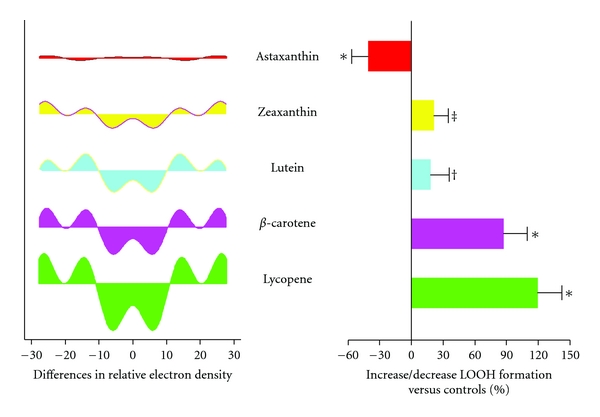
Antioxidant activity depends on molecular structure and localization (“Alignment”) in cellular/mitochondrial Membrane. Extent of deviation in the X-ray crystallographic pattern correlates with an increase in lipid peroxide formation. Correlation between membrane structure changes and LOOH formation (LOOH, lipid peroxide). Differences in relative electron density as a function of treatment with various carotenoids in POPC membranes containing a C/P mole ratio of 0.2. For the peroxidation study, various carotenoids (10 *μ*M) were incorporated into DLPC membranes and underwent lipid peroxidation at 37°C for 48 h. Expressed as percent increase or decrease in LOOH (lipid peroxide) formation compared to controls containing no carotenoids. *P < 0.001 versus control; ^‡^ *P* < 0.01 versus control; ^†^ *P* < 0.05 versus control; *n* = 5 ~ 6. (POPC, 1-palmitoyl 2-oleoyl-3-sn-glycerophosphatidylcholine), adapted from McNulty et al. [46].

The naïve assumption is that all antioxidants are essentially the same. Nothing can be further from the truth. All antioxidants are not the same: they may work in substantially different ways (chain-breaking versus singlet oxygen quenching, e.g.), and in different locations (e.g., in the bilayer membrane versus the cytoplasm) and *very small* differences in molecular structure can have profound influence on biological activity. In the carotenoid family, for example, distinct effects occur in lipid peroxidation due to membrane structure changes. *β*-carotene, which misaligns when localized in the bilayer membrane is highly disruptive structurally and can be functionally pro-oxidant when compared to structurally similar members of the family that align completely (Figure 2) [46]. These contrasting effects of carotenoids on lipid peroxidation may explain the clinical outcomes observed in various randomized trials. 

## 10. Understanding Antioxidant Mode of Action Is Critically Important

Further, lack of understanding of the mode of action [66] has led to erroneous clinical designs and patient selection. Little attempt was made to scale the antioxidant potential of the therapy to the underlying oxidative stress. Atherosclerosis is a multifactorial disease and LDL is oxidized by all major cells of the arterial wall during the development of atherosclerosis via more than one mechanism. The various LDL oxidation pathways produce several lipid peroxidation products such as isoprostanes from several fatty acids, oxysterols from unesterified and esterified cholesterol, hydroxy fatty acids, lipid peroxides, and aldehydes. Intervention trials should be accompanied by measurements of one or more of these relevant biomarkers at intervals during the study and the correlation of the biomarkers to the therapeutic intervention needs to be established. In addition to the markers in use for lipid peroxidation, there is a need to include markers for endothelial dysfunction, monocyte adhesion, macrophage uptake of lipoproteins, thrombotic, and inflammatory processes [67].

**Figure 3:**
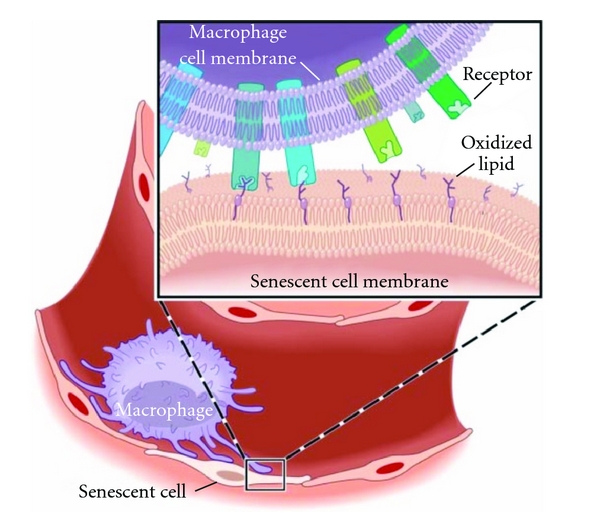
Oxidized phospholipids protrude from cell membranes in senescent endothelial cells forming oxidized lipid “whiskers.” Schematic representation of the lipid whisker model. Cell membranes of senescent endothelial cells can possess oxidized phospholipids with protruding *sn*-2-oxidized fatty acid acyl chains into the extracellular space. This conformation renders them accessible to interact with scavenger receptors and other pattern recognition receptors on the surface of platelets or probing macrophages of the circulatory and the immune system, adapted from [51].

Our recognition of the connection between oxidative stress, inflammatory signaling, and such critical manifestations of atherosclerotic cardiovascular disease as atherothrombosis is growing. The cellular membranes of endothelial cells can possess oxidized phospholipids with protruding *sn*-2-oxidized fatty acid acyl chains into the extracellular space. This conformation renders them accessible to interact with scavenger receptors and other pattern recognition receptors on the surface of platelets or probing macrophages of the circulatory and the immune system (Figure 3) [51].

**Figure 4:**
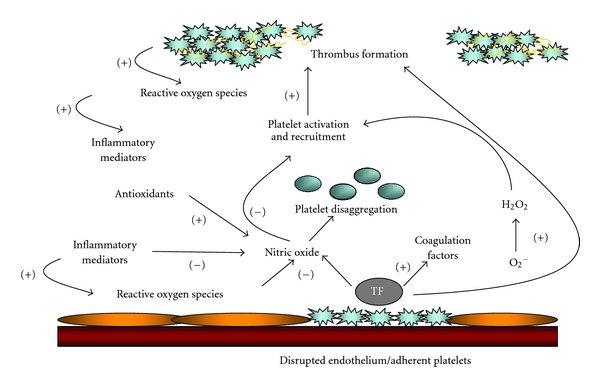
Oxidative stress mediates abnormal platelet function and dysfunctional endothelium-dependent vasodilation. Oxidative stress is an important mediator of both abnormal platelet function and dysfunctional endothelium-dependent vasodilation in the setting of cardiovascular disease. Superoxide anion is an important source of oxidative stress, has direct effects, and limits the biological activity of NO. Excessive vascular superoxide production drives further platelet activation and recruitment leading to greater thrombus formation. The occurrence of superficial intimal injury caused by endothelial denudation and deep intimal injury caused by plaque rupture expose collagen and Tissue Factor (TF) to platelets. Local platelet activation stimulates further thrombus formation and additional platelet recruitment by supporting cell-surface thrombin formation and releasing potent platelet agonists such as adenosine diphosphate (ADP), serotonin, and thromboxane A2. A thrombus forms as platelets aggregate via the binding of bivalent fibrinogen to GP IIb/IIIa. Platelet NO release influences platelet recruitment to the growing thrombus and impaired platelet-derived NO release is likely associated with acute coronary and stroke syndromes. Antioxidants may indirectly inhibit platelets through scavenging of reactive oxygen species, many of which alter platelet function. Despite the different subcellular locations of water- and lipid-soluble antioxidants, these antioxidant pathways in platelets are closely linked. Antioxidants may also indirectly inhibit platelets through the metabolism of reactive oxygen species, many of which alter platelet function. Inflammation is linked with the evolution of cardiovascular disease and acute coronary syndromes, adapted from [15].

Oxidative stress is an important mediator of both abnormal platelet function and dysfunctional endothelium-dependent vasodilation in the setting of cardiovascular disease. Superoxide anion is an important source of oxidative stress, has direct effects, and limits the biological activity of NO. Excessive vascular superoxide production drives further platelet activation and recruitment leading to greater thrombus formation. The occurrence of superficial intimal injury caused by endothelial denudation and deep intimal injury caused by plaque rupture expose collagen and tissue factor [62] to platelets. Local platelet activation stimulates further thrombus formation and additional platelet recruitment by supporting cell-surface thrombin formation and releasing potent platelet agonists such as adenosine diphosphate (ADP), serotonin, and thromboxane A2. A thrombus forms as platelets aggregate via the binding of bivalent fibrinogen to GP IIb/IIIa. Platelet NO release influences platelet recruitment to the growing thrombus and impaired platelet-derived NO release is likely associated with acute coronary and stroke syndromes (Figure 4) [15]. Thus, antioxidants may indirectly inhibit platelets through scavenging of reactive oxygen species, many of which alter platelet function. Despite the different subcellular locations of water- and lipid-soluble antioxidants, these antioxidant pathways in platelets are closely linked. Antioxidants may also indirectly inhibit platelets through the metabolism of reactive oxygen species, many of which directly alter platelet function.

Oxidative stress and inflammation are intimately linked with both the evolution of cardiovascular disease and acute coronary syndromes. It should be no surprise that ox-LDL levels show a significant positive correlation with the severity of acute coronary syndromes and that the more severe lesions also contain a significantly higher percentage of ox-LDL-positive macrophages. Such observations suggest that increased levels of ox-LDL relate to plaque instability in human coronary atherosclerotic lesions [68, 69].

## 11. Dose-Response Documentation Lacking

Implicit in the randomized trials is that the dose of antioxidant tested (usually vitamin E), effectively suppressed oxidative stress but this was never determined [70]. In fact, studies suggest that the dosages of the compounds tested and/or the duration of therapy was not adequate. In one time-course study, maximum suppression of plasma F2-isoprostane concentrations did not occur until 16 weeks of supplementation. In the dose-ranging study there was a linear trend between the dosage of vitamin E and percentage reduction in plasma F2-isoprostane concentrations which reached significance at doses of 1600 IU (35 ± 2%, *P* < 0.035) and 3200 IU (49 ± 10%, *P* < 0.005) [70]. Whether such dosages in human subjects would be safe and if the compound was administered early enough in the lifecycle of the disease process are other essential considerations.

## 12. Safety Now an Overarching Issue

Safety is the overarching issue in drug development today, but little was done historically to determine if the antioxidant being tested for an inflammatory-mediated cardiovascular manifestation is safely tolerated at the levels required to provide therapeutic relief [71, 72]. Since the high standards of chemistry, manufacturing, and controls (CMC) required for pharmaceutical drugs are unlikely to be applied to nutraceutical or dietary-supplement-type products, many of the questions regarding safety and efficacy of antioxidants will most likely be answered in the future related to the development of proprietary prodrugs seeking regulatory approval. These antioxidant drug candidates, incidentally, will most likely have greater druglikeness and bioavailability.

## 13. Conclusion

Despite the lack of significant randomized clinical trial data supporting their use, more than $20b is still being spent annually on the antioxidant vitamins A, C, and E with more than 6 million tons of the latter projected to be consumed annually on a global basis [73]. My belief is that antioxidants because of their provenance as “natural products” or “nutritional supplements” and their presumption of safety and efficacy generated from the results of epidemiologic and observational studies early-on, have not been subjected to the same stringent developmental requirements that are applied to new pharmaceutical drug candidates. Biologically active compounds, formulated properly, administered in appropriate amounts for an appropriate duration to the right patients will be required to achieve all the requirements that truly define therapeutic success.

## Figures and Tables

**Table 1:** Human proof-of-concept studies demonstrating effectiveness of various antioxidant regimens on cardiovascular endpoints.

Study	*n*	Intervention	Endpoint	Antiox Rx results	Placebo results	*P* value
SPACE [16]	196	Vit E 800 IU/day	Composite endpoint^1^	16%	33%	= 0.014

IEISS [17]	125	Vit A 50,000 IU/day, Vit C 1,000 mg/day, Vit E 400 mg/day, *β*-carotene 25 mg/day	Individual component scores^2^	20.6	30.6	“Sig. less”

VCE-MI [18]	61	Vit C&E 600 mg/day	SAECG^3^	No Δ	“Sig. Δ”^3^	<0.002

PART [19]	101	Probucol 1,000 mg/day	Restenosis p PCI	23%	58%	= 0.001

ASAP [20]	520	d-alpha-tocopherol 91 mg, Vit C 250 mg/day	Carotide IMT	0.011 mm/year-1	0.020 mm/year-1	= 0.008

MVP [21]	317	*β*-carotene 30,000 i.u., Vit C 500 mg/day, Vit E 700 IU/day, Probucol 500 mg/day	Restenosis p PCI	28.9%	38.9%	“Sig. less”

SPACE: Secondary Prevention with Antioxidants of Cardiovascular disease in Endstage renal disease; IEISS: Indian Experiment of Infarct Survival Study; VCE-MI: Vitamins C&E on Myocardial Infarction; PART: Probucol Angioplasty Restenosis Trial; ASAP: Antioxidant Supplementation in Atherosclerosis Prevention; MVP: Multivitamins and Probucol Study Group.

^1^Composite Endpoint: myocardial infarction (fatal and nonfatal), ischemic stroke, non-AV fistular peripheral vascular disease, and unstable angina.

^2^Individual Component Scores: mean infarct size (creatine kinase and creatine kinase-MB gram equivalents), serum glutamic-oxaloacetic transaminase, cardiac enzyme lactate dehydrogenase increased, and, QRS score in the electrocardiogram.

^3^SAECG: Signal-average electrocardiogram components consist of increase in mean QRS and low-amplitude (<40 microV) signal durations, a decrease in the root-mean-square voltage of the last 40 ms of the QRS complex.
